# Bacterial profile, antimicrobial resistance patterns and associated factors among cancer patients at Hawassa University Comprehensive Specialized Hospital, Sidama region, Southern Ethiopia

**DOI:** 10.1186/s12879-026-13061-8

**Published:** 2026-04-10

**Authors:** Alemitu Beyene, Demissie Assegu Fenta, Yared Merid

**Affiliations:** College of Medicine and Health Sciences, Hawassa University, Hawassa, Ethiopia

**Keywords:** Bacterial profile, Antibiotic resistant, Cancer patients, Hawassa University

## Abstract

**Background:**

Infectious diseases are the leading cause of morbidity and mortality among cancer patients due to impaired immunity and the immunosuppressive effect of cancer treatment. Therefore, the aim of this study was to determine the bacterial profile, antimicrobial resistance patterns, and factors associated among cancer patients at Hawassa University Comprehensive Specialized Hospital, Sidama region, Southern Ethiopia.

**Methods:**

A cross-sectional study was conducted on 266 bacterial infection suspected cancer patients from January 1 to June 30, 2024. Socio-demographic and clinical data were collected using a structured questionnaire. Blood, urine, sputum, and swab samples were collected and transported to the Sidama Region Public laboratory, microbiology laboratory for bacterial investigation. Biochemical and antimicrobial susceptibility testing was done using standard bacteriological techniques. Data were analyzed using SPSS version 28. Bivariate and multivariable logistic regression was done to assess associated factors and a p-value of < 0.05 with 95% CI were considered statistically significant.

**Result:**

Of the 266 samples cultured, 56 (21.1%) (95% CI = 16.2–25.9) were culture positive for bacterial infection. CoNS, 18 (32.1%) was the predominant isolate, followed by *E. coli*, 11 (19.6%) and *K. pneumonia*, 9 (16.1%). Gram-positive bacteria showed high resistance to nitrofurantoin, 23 (88.5%), tetracycline, and penicillin, 22 (84.6%) each. Similarly, gram negative bacteria showed high resistance to nitrofurantoin, 23 (76.7%) and tetracycline, 21 (70.0%). The overall prevalence of MDR organisms in this study was 46 (82.1%). All *S. aureus* and *K. oxytoca* isolates were MDR. Being a rural resident (AOR = 0.47; 95% CI = 0.237–0.918; *P* = 0.027), stage I cancer (AOR = 0.08; 95%CI = 0.007–0.884, *P* = 0.039) and patients with a cancer in progression, (AOR = 2.52; 95%CI = 1.011–6.298, *P*=0.047) were statistically associated with bacterial infection.

**Conclusion:**

In the current study, the prevalence of bacterial infection among cancer patients was high (21.1%). CoNS were the most dominant isolates among cancer patients. High resistance of the isolates to commonly used antibiotics; nitrofurantoin, tetracycline, and penicillin were observed. Rural residence, stage I cancer, and progressive cancer showed significant associations with bacterial infections in cancer patients. Thus, the treatment of cancer patient’s bacterial infection should depend on an antimicrobial susceptibility test. Moreover, evaluation of bacterial infection risk factors and tracking of antibiotic resistance are crucial in the controlling of bacterial infection and drug resistance among cancer patients.

**Clinical trial:**

Not applicable.

**Supplementary Information:**

The online version contains supplementary material available at 10.1186/s12879-026-13061-8.

## Introduction

Cancer patients are more vulnerable to infections compared to general populations, due to basic immunodeficiency and cancer interventions in the form of chemotherapy, surgery, and radiotherapy [1, 2]. Bacterial infections are the most leading cause of morbidity and mortality among cancer patients [3–5]. These infections commanly originate from the patient’s normal skin flora, implanted medical devices, or the hospital environment, including healthcare workers, air, and medical equipment [6]. Bloodstream infections (BSI) [7] and Urinary Tract Infection (UTI) are the most common bacterial infections in the cancer patients [8] with coagulase-negative staphylococci (CoNS), *Staphylococcus aureus* (*S. aureus*) *Escherichia coli* (*E. coli*), *Klebsiella pneumonia* (*K. pneumonia*) and *Citrobacter spp* being the predominant pathogens [9–11].

Global cancer deaths have risen by over 70% in the past 30 years [12]. It was the second leading cause of global death, accounting for 10 million deaths in 2020; of them, 70% occurred in low- and middle-income countries [13]. Antimicrobial resistant (AMR) pathogens infection is a major factors in the rising of cancer patient’s death, morbidity, and medical expenses [14]. Actually, AMR bacterial infection is a main global public health problem and was responsible for approximately 1.27 million deaths in 2019 [15]. Cancer patients acquiring AMR bacterial infections due to regular hospital attendance and admissions, prophylactic antibiotic use, surgical procedures and catheterization [14, 16]. In particular, BSI and UTI among cancer patients are associated with multidrug resistant (MDR) Gram-negative bacterial infection, which contribute to increased patient death [4, 17]. The burden of MDR bacterial infections among cancer patients is especially high in developing countries. For example, in India, about 73% of carbapenem-resistant bacteria were isolated from blood cancer patients [18]. Also in Uganda the authors reported 85% different MDR Enterobacteriaceae isolated from BSI in cancer patients [19]. Moreover, in the Napel study, about 89% of bacterial isolates from UTI among cancer patients were MDR [20]. Similarly, Ethiopia shares 33.3% to 88.3% of MDR bacterial isolated among cancer patients with high rate (84.1%) of ESBL producer bacteria [10, 11, 21–23]. High resistance of isolates were observed against ciprofloxacin, trimethoprim- sulfamethoxazole, penicillin, amoxicillin, and tetracycline antibiotics, with high number of MDR Gram-negative bacteria (88.3%) [22, 24].

Despite this burden, infection treatment in cancer patients often relies on empirical use of broad spectrum antibiotics and a combination drugs rather than culture guided therapy particularly in developing countries, which increases AMR development and MDR risk [8, 25, 26]. This is the major challenge in the controlling of MDR pathogens within this group and in the wider community and also increases hospitalization duration and treatment costs [27–29]. The prevalence and antibiotic susceptibility patterns of pathogens in cancer patients vary across populations and geographic regions. Thus accurate laboratory identification and adherence to treatment guidelines are very essential [21]. In Ethiopia, data on the bacterial profile and antimicrobial susceptibility patterns among cancer patients are limited, particularly in the southern region. The few existing studies have primarily focused on bloodstream infections (BSIs) [22–24], with little attention given to other types of infections such as urinary tract infections (UTIs), respiratory infections and local infection. Therefore, this study aims to provide updated and locally relevant data on the bacterial profile, antimicrobial resistance patterns, and associated factors among cancer patients in the southern Ethiopia.

## Materials and methods

### Study area, design, and period

Hospital-based cross-sectional study was conducted among at, Hawassa University Comprehensive Specialized Hospital (HUCSH) Sidema region, Ethiopia from January 1 to June 30/2024. Hawassa is the capital city of the region and located 275 km away from Addis Ababa, the capital city of Ethiopia. The altitude of the town is 1697 m above sea level with mean annual temperature and rainfall of 20.9 °C and 997.6 ml respectively. It is the only Hospital which provides cancer treatment and management service in southern part of Ethiopia including some Oromia region, and provides chemotherapy and radiation treatment for more than 800 new cases per year. Besides this, the hospital is providing services in terms of internal medicine, Surgery, Gynecology/Obstetrics, Pediatrics, dentistry, Ophthalmology, Dermatology, and Psychiatry.

### Study population

All cancer patients attending the cancer center of Hawassa University Comprehensive Specialized Hospital (HUCSH) during the study period.

### Study subject

All Cancer patients who attended the cancer center of HUCSH for medical services and were suspected of having bacterial infections during the study period.

### Inclusion and exclusion criteria

All cancer patients who provided consent to participate were included in the study, while those who had received antibiotic treatment within the last 14 days were excluded from this study.

### Sample size and sampling technique

The Sample size was calculated by using single population proportion formula, considering the following assumptions (Z=standard normal distribution with a confidence interval of 95%=1.96, the P = prevalence of 19.4% from previous study conducted in cancer patients in Gondar [11], Ethiopia, and 5% Marginal error). Finally, by considering the 10% non-response rate the sample size calculated was 266. A systematic random sampling technique was applied to select 266 respondents for interview. According to data found from the database of HUCSH the average daily cancer patient with infection suspected at HUCSH cancer center was 4. The study period was expected to take four months which contain 88 work days and 4 times 88 gives 352. To choose the first participant, the K interval was calculated. After the K interval was calculated, the first individual was selected by the lottery method and the others at a regular interval, K = 352/266 = 1.3 ≈ 2.

### Operational definition

Multidrug resistance (MDR): a species of microorganisms that is resistant to at least one agent in ≥ 3 antimicrobial categories [30].

### Socio-demographic and clinical data collection

All relevant socio-demographic and clinical data were collected by trained nurses through face-to-face interviews with the patient under physician supervision using a structured questionnaire during the clinical examination (File S1).

### Sample collection and processing

#### Blood sample collection and processing

Beflection, skin antisepsis and bottle disinfection were performed using 70% alcohol. Then, 5-10mL blood samples were aseptically collected from peripheral veins of each patient with febrile for culture process. The collected sample was inoculated to tryptone soya broth (Oxoid Ltd, UK) and transported to Sidama Region Public laboratory, Microbiology lab for investigation. The bottle was incubated at 35–37 °C for 7 days and media had been inspected daily for colony growth. Bottle that showed signs of growth with turbidity was sub cultured to chocolate agar, blood agar (in candle jar) and MacConkey (aerobically at 35–37 °C) [ore blood col^31^].

#### Urine sample collection and processing

Study participants’ was oriented on how to collect clean-catch midstream urine samples. Ten milliliter of urine samples were aseptically collected from each patient with a sterile urine cap [32]. Then the collected sample was transported to Sidama Region Public laboratory, Microbiology laboratory within 2 h for further investigation. Then the urine sample was inoculated onto MacConkey agar plate and blood agar (Oxoid, Basingstoke, and Hampshire, England) medium with calibrated sterile wire loop of 1 µL capacity. All the media were incubated at overnight at 37 °C. Significant bacteriuria growth was defined as colony count ≥ 10^5^ CFU/mL urine [33, 34].

#### Sputum collection and processing

Sputum collection: the patient was first instructed how to bring a sputum sample by laboratory technologist. Then spot sputum samples were collected with one-hour interval using dry, sterile, leak-proof, translucent, and screw-capped plastic containers with a capacity of 30 ml. The quality of sputum were checked macroscopically for colour, volume, viscosity, odour, and Gram’s stain was performed to evaluate the acceptability of sputum based on Bartlett’s criteria [35].

The purulent part of accepted sputum sample was inoculated to blood agar, MacConkey agar and chocolate agar with the sterile wire loop. In order to get a single pure colony, the samples were streaked into four quadrants of the plate with flaming the loop in between each spread. The inoculated MacConkey agar plates were incubated aerobically at 37 °C for 24 h. However, blood agar and chocolate agar were inoculated using 5–10% CO_2_ generating candle jar at 37 °C for 24 h. After 24 hours’ incubation, the plate was examined for growth. Pure colonies were selected and sub-cultured to nutrient broth [36].

#### Swab sample collection and processing

Based on clinical manifestation other swab samples, wound, ear discharge were collected after site sample collected cleaned by sterile saline using sterile saline moistened cotton swab by trained nurse or physician. Then collected were transported to Sidama Region Public laboratory, Microbiology within two hours using Amies transport medium. The swabs were inoculated to 5% sheep’s blood agar plates (blood agar base Oxoid UK), MacConkey agar and chocolate agar by rolling the swab over a small area of the plate and streaking the sample using a sterile loop. The plate was incubated for 24 h at 37 °C in a candle jar, which can provide an atmosphere of 5% CO_2_ [11, 36].

#### Bacterial identification

Pure isolates of bacterial pathogens were preliminarily characterized by colony morphology, gram stain and hemolytic reactions on blood agar plates. Identification of bacteria down to species level was done by biochemical tests such as catalase, coagulase, bacitracin (30 µg) test and CAMP test for Gram-positive identification, while oxidase, indole production, urease, citrate utilization, lysine decarboxylation, carbohydrate fermentation, gas production, H_2_S production and motility for Gram-negative bacterial identification [37].

#### Antimicrobial susceptibility testing

Antimicrobial susceptibility tests were performed for all isolated bacteria by disc diffusion method using Mueller Hinton blood agar and 5% sheep blood complemented muller Hinton agar according to CLSI 2024 guidelines [38] and on prevailing local prescribing practices. Based on this guideline, ceftriaxone (30 µg), clindamycin (2 µg), erythromycin (15 µg), penicillin (10 units), ampicillin (30 µg), chloramphenicol (30 µg), ciprofloxacin (5 µg) tetracycline (30 µg), nitrofurantoin (30 µg), amikacin (30 µg), (cefoxitin (30 µg), Trimethoprim/sulfamethoxazole (25 µg) and meropenem (10 µg) antibiotics were selected for the study. Finally, the zone of inhibition was measured with a ruler and interpreted according to the CLSI 2023 guideline value.

#### Data quality control

English version of questionnaires were translated to Amharic, Sidama Afo and Afan Oromo langues and then back to English to check for consistency before data collection. Finally, the Amharic, Sidama Afo and Afan Oromo version were used. Prior to actual data collection, the quality of data were assured by pre-testing questionnaires on 13 participants at Adare General Hospital, Hawassa for assessing its clarity and amendments. Orientation was given to data collectors before data collection.

To maintain the quality of the work from sample collection up to final laboratory identification and data management the standard operating procedure of sample collection and laboratory analysis was strictly followed. All the equipment’s and reagents were checked for their functionality. The sterility of prepared culture media was checked by incubating the 5% of prepared media in 5% CO_2_ enriched atmosphere at 37 °C for 24 hours and observed for the presence of any colony growth. In addition, the abilities of the prepared media supporting the growth of organism was checked by inoculating control strains *E. coli* ATCC 25922, *P. aeruginosa* ATCC 27853, and *S. aureus* ATCC 25923 from Ethiopian public health institute (EPHI). After collection, all data including laboratory results were checked for completeness and relevance’s each day.

#### Data analysis

All questionnaires for this study were checked visually, coded and uploaded to kobotoolbox humanitarian application for data collection purpose. After data were collected from all study participants, they were exported to SPSS version 28 windows program for analysis. To explain the study population in relation to relevant variables descriptive statistics (frequencies, mean, standard deviation and percentage) was used. Bivariate logistic regression analysis was used to determine the associated risk factors. For those variables, which p-value < 0.25 in the bivariate, the analysis was further entered into the multivariable logistic regression model. Associations between dependent and independent variables were assessed and its strength was described using odds ratios at 95% confidence intervals. P-value < 0.05 was accepted as statistically significant, and finally, the result was presented using statements, tables and graph.

## Result

### Socio-demographic characteristics of study participants

Out of 266 cancer patients enrolled in this study, 145 (54.5%) were females. The participants’ mean age was 48.8 ± 14.97 year, with age range 17–86 years. More than half of the, 137 (51.5%) were from rural areas. The majority of 232 (87.2%) were married, and 117 (44.0%) of participants had no formal education. Regarding occupation, 92 (34.6%) of were household, and 128 (48.1%) of participants earned a monthly income of 1501–3000 ETB (Table 1).

### Clinical characteristics of study participants

Regarding clinical variables, 11 (4.1%) of the participants had a family history of cancer. Majority, 161 (60.5%) of the participants were inpatient. Breast cancer was the most common type, 73 (27.4%), followed by gastric cancer, 33 (12.4%). A total of 127 (47.7%) participants were in stage IV cancer, and 204 (76.7%) had started cancer therapy, predominantly chemotherapy (191; 71.8%). Cancer progression was observed in 223 (83.8%) cases (Table 1).

**Table 1:** Frequency of socio-demographic and clinical characteristics of cancer patients at HUCSH, Sidama region, Southern Ethiopia, 2024

Socio-demographic variables	Categories	Frequency	Percentage
Sex	Male	121	45.5
	Female	145	54.5
Age	< 25	13	4.9
	25–35	43	16.2
	36–45	66	24.8
	46–60	96	36.1
	> 60	48	18.0
Residence	Urban	129	48.5
	Rural	137	51.5
Marital status	Single	25	9.4
	Married	232	87.2
	Divorced	9	3.4
Education status	No formal	117	44.0
	Elementary	49	18.4
	High school	77	29.1
	Diploma & above	23	8.6
Occupation	Government	24	9.0
	Private	46	17.3
	Farmer	64	24.1
	House hold	92	34.6
	No work	40	15.0
Income	> 4500	44	16.5
	< 1500	56	21.1
	1501–3000	128	48.1
	3001–4500	38	14.3
Smoking	Yes	10	3.8
	No	256	96.2
Clinical variables			
Family history of cancer	Yes	11	4.1
	No	255	95.9
History of previous hospitalization	Yes	39	14.7
	No	227	85.3
Patient setting	Inpatient	161	60.5
	Outpatient	105	39.5
Type of cancer	Breast	73	27.4
	Colon	18	6.8
	Gall bladder	13	4.9
	Cervical	23	8.6
	Esophageal	26	9.8
	Pancreatic	11	4.1
	Gastric	33	12.4
	Hematology	5	1.9
	Lung	9	3.4
	Prostatic	5	1.9
	Others	50	18.8
Stage of cancer	I	13	4.9
	II	56	21.1
	III	55	20.7
	IV	127	47.7
	Unknown	15	5.6
Cancer therapy started	Yes	204	76.7
	No	62	23.3
Type of cancer therapy	Surgery	27	10.2
	Chemotherapy	191	71.8
	Follow up	48	18.0
Duration of therapy	≥ 1 year	27	10.2
	< 1 year	191	71.8
	Follow up	48	18.0
Surgical incision history	Yes	66	24.8
	No	200	75.2
History of catheterization	Yes	25	9.4
	No	241	90.6
Duration of catheter	Previous	11	4.1
	Current	14	5.3
	No catheter	241	90.6
Febrile	Yes	19	7.1
	No	257	92.9
Cancer progression	Yes	223	83.8
	No	43	16.2

### Bacterial profiles of cancer patients

A total of 266 culture specimens were collected, with one type of sample obtained from each study participant. Urine specimens were the most frequent, 208 (78.2%), followed by sputum 29 (10.9%). Of these specimens, 56 (21.1%) had bacterial growth. The majority of isolates were recovered from urine 42 (75.0%) and sputum 8 (14.3%), with a small number isolate from ear discharge 1 (0.4%). Among the total 56 isolates, Gram negative bacteria (GNB) accounted for 30 (54.0%), which was higher than Gram positive bacteria (GPB), 26 (46.0%). Overall, CoNS 18 (32.1%) was the predominant bacterial isolate, followed by *E. coli* 11 (19.6%) and *K. pneumonia* 9 (16.1%) (Table 2).

**Table 2:** Distribution of bacterial isolates from different clinical specimens among cancer patients at HUCSH, Sidama region, Southern Ethiopia, 2024

Bacterial isolate	Clinical specimen *n* (%)					
	Urine 208 (78.2)	Sputum 29 (10.9)	Blood 26 (9.8)	Wound swab 2 (0.8)	Ear discharge 1 (0.4)	Total 266 (100)
CoNS	17	1	0	0	0	18 (32.1)
*S. aureus*	4	0	0	1	0	5 (8.9)
*S. agalactiae*	1	1	1	0	0	3 (5.4)
Subtotal of gram positive	**22**	**2**	**1**	**1**	**0**	**26 (46.0)**
*E. coli*	10	0	0	1	0	11 (19.6)
*K. pneumonia*	5	2	1	0	1	9 (16.1)
*K. oxytoca*	4	0	1	0	0	5 (8.9)
*P. valgaris*	0	1	0	0	0	1 (1.8)
*C. jillenii*	1	1	0	0	0	2 (3.6)
*P. stuartii*	0	1	0	0	0	1 (1.8)
*M. morgarin*	0	1	0	0	0	1 (1.8)
Subtotal of gram negative	**20**	**6**	**2**	**1**	**1**	**30 (54.0)**
Grand total n (%)	**42 (75.0)**	**8 (14.3)**	3 (5.2)	**2 (3.6)**	**1 (1.8)**	**56 (100)**

Key; CoNS: Coagulase-negative staphylococci

### Antimicrobial resistance patters of bacterial among cancer patients

In this study gram-positive bacteria showed high resistance to nitrofurantoin, 23 (88.5%) as well as to tetracycline and penicillin, 22 (84.6%) each. In contrast, relatively low resistance was observed to clindamycin, 10 (38.5) and erythromycin 11 (42.3%). The most dominant organism, CoNS showed high resistance to nitrofurantoin, 16 (88.9%) and Trimethoprim/sulfamethoxazole 13 (72.2%) antibiotics, but low resistance to clindamycin 7 (38.9%). The second prevalent GPB *S. aureus*, showed 100% resistance to Trimethoprim/sulfamethoxazole, tetracycline, penicillin and cefoxitin, while remaining fully sensitive to erythromycin. *Streptococcus agalactiae* (*S. agalactiae*) was 100% resistant to ciprofloxacin, tetracycline, and penicillin nitrofurantoin antibiotics (Table 3).

**Table 3:** Antimicrobial resistance pattern of GPB isolated from cancer patients at HUCSH, Sidama region, Southern Ethiopia, 2024

Antibiotics	Pattern	Gram positive bacteria			Total *n*/26 (%)
		CoNS (*n* = 18)	*S. aureus* (*n* = 5)	Beta hemolaytic streptococcus (*n* = 3)	
Cotrimozole	S	5 (28.8)	0	1 (33.3)	6 (23.1)
	R	13 (72.2)	5 (100)	2 (66.7)	20 (67.9)
Ciprofloxacin	S	8 (44.4)	4 (80.0)	0	12 (46.2)
	R	10 (55.6)	1 (20.0)	3 (100)	14 (53.8)
Tetracycline	S	4 (22.2)	0	0	4 (15.4)
	R	14 (77.8)	5 (100)	3 (100)	22 (84.6)
Nitrofurantoin	S	2 (11.1)	1 (20.0)	0	3 (11.5)
	R	16 (88.9)	4 (80)	3 (100)	23 (88.5)
Erythromycin	S	7 (38.9)	5 (100)	1 (33.3)	13 (50.0)
	R	9 (50.0)	0	2 (63.7)	11 (42.3)
	I	2 (11.1)			2 (7.7)
Clindamycin	S	11 (61.1)	4 (80.0)	1 (33.3)	16 (61.5)
	R	7 (38.9)	1 (20.0)	2 (63.7)	10 (38.5)
Penicillin	S	4 (22.2)	0	0	4 (15.4)
	R	14 (77.8)	5 (100)	3 (100)	22 (84.6)
Cefoxitin	S	6 (33.3)	0	NA	6/23 (26.1)
	R	12 (63.7)	5 (100)		17/23 (73.9)

Key; CoNS: Coagulase-negative staphylococci, R: Resistance, S: Sensitivity, I: Intermediate

On the other hand, gram-negative bacteria also showed high resistance to nitrofurantoin, 23 (76.7%) and tetracycline, 21 (70.0%), while showing low resistance to meropenem, 2 (6.7%) and amakacin and chloramphenicol, 4 (13.3%) each. *E. coli*, the most prevalent among GNB, showed high-resistant to ampillicin, 10 (90.0%) and tetracycline, 9 (81.8%). However, it showed low resistance to meropenem, 1 (9.1%), as well as to gentamycin, chloramphenicol, and amikacin, 2 (18.2%) each. *K. pneumonia* showed high resistance to Nitrofurantoin 6 (63.6%) and relatively low resistance to meropenem, gentamycin, chloramphenicol, and amikacin, 1 (11.1%) each. *Klebsiella oxytoca* (*K. oxytoca*) showed 100% resistance to nitrofurantoin, and was 100% sensitive to meropenem and amikacin antibiotics (Table 4).

**Table 4:** Antimicrobial resistance patterns of GNB isolated from cancer patients at HUCSH, Sidama region, Southern Ethiopia, 2024

Antibiotics	Pattern	Gram negative bacteria							Total *n*/30 (%)
		*E. coli*	*K. pneumoniae*	*K. oxytoca*	*C. jillenii*	*P. valgaris*	*P. stuartii*	*M. morgarin*	
Trimethoprim/sulfamethoxazole	S	4 (36.4)	4 (44.4)	1 (20.0)	0	1 (100)	1 (100)	1 (100)	12 (40.0)
	R	7 (63.6)	5 (55.6)	4 (80)	2 (100)	0		0	18 (60.0)
Ciprofloxacin	S	7 (63.6)	4 (44.4)	3 (60.0)	0	1 (100)	1 (100)	1 (100)	17 (56.7)
	R	4 (36.4)	5 (55.6)	2 (40.0)	2 (100)	0	0	0	13 (43.3)
Tetracycline	S	2 (18.2)	4 (44.4)	1 (20.0)	0	1 (100)	0	1 (100)	9 (30.0)
	R	9 (81.8)	5 (55.6)	4 (80)	2 (100)	0	1 (100)	0	21 (70.0)
Nitrofurantoin	S	4 (36.4)	3 (33.4)	0	0	0	0	0	7 (23.3)
	R	7 (63.6)	6 (63.6)	5 (100)	2 (100)	1 (100)	1 (100)	1 (100)	23 (76.7)
Ceftriaxone	S	7 (63.6)	4 (44.4)	1 (20.0)	1 (50.0)	1 (100)	0	1 (100)	15 (50.0)
	R	4 (36.4)	5 (55.6)	4 (80)	1 (50.0)	0	1 (100)	0	15 (50.0)
Gentamycin	S	9 (81.8)	8 (88.9)	1 (20.0)	0	1 (100)	1 (100)	1 (100)	21 (70.0)
	R	2 (18.2)	1 (11.1)	4 (80)	2 (100)	0	0	0	9 (30.0)
Ampillicin	S	1 (9.1)	5 (55.6)	1 (20.0)	1 (50.0)	0	1 (100)	0	9 (30.0)
	R	10 (90.0)	4 (44.4)	4 (80)	1 (50.0)	1 (100)	0	1 (100)	21 (70.0)
Chloramphenicol	S	9 (81.8)	8 (88.9)	4 (80)	2 (100)	1 (100)	1 (100)	1 (100)	26 (87.7)
	R	2 (18.2)	1 (11.1)	1 (20.0)	0	0	0	0	4 (13.3)
Amikacin	S	9 (81.8)	8 (88.9)	5 (100)	1 (50.0)	1 (100)	1 (100)	1 (100)	26 (87.7)
	R	2 (18.2)	1 (11.1)	0	1 (50.0)	0	0	0	4 (13.3)
Meropenem	S	10 (90.9)	8 (88.9)	5 (100)	2 (100)	1 (100)	1 (100)	1 (100)	28 (93.3)
	R	1 (9.1)	1 (11.1)	0	0	0	0	0	2 (6.7)

Key; R: Resistance, S: Sensitivity, I: Intermediate

### Multiple drug resistance (MDR)

In this study, the overall prevalence of MDR was 46 (82.1%). In contrast, 2 (3.6%) of the bacterial isolates were sensitive to all antibiotics tested, and none of the pathogens showed resistant to all antibiotic classes. *S. aureus*, *S. agalactiae*, *K. oxytoca*,* Citrobacter jillenii* (*C. jillenii*) showed 100% MDR. The most dominant organisms, CONS showed 16 (88.9%) MDR. Additionally, 8 (72.7%) of *E. coli* and 5 (55.6%) of K. *pneumoniae* isolates were classified as MDR (Table 5).

**Table 5:** Multiple drug resistance patterns of bacteria isolated from cancer patients at HUCH, Sidama region, Southern Ethiopia, 2024

Bacterial isolate	Antibiogram pattern									Total MDR *n* (%)
	R0	R1	R2	R3	R4	R5	R6	R7	R8	
CONS (*n* = 18)	0	0	2 (11.1)	2 (11.1)	2 (11.1)	4 (22.2)	4 (22.2)	4 (22.2)	0	16 (88.9)
*S. aureus* (*n* = 5)	0	0	0	0	0	1 (20.0)	3 (60.0)	1 (20.0)	0	5 (100)
*S. agalactiae* (*n* = 3)	0	0	0	0	0	2 (66.7)	1 (33.3)	0	0	3 (100)
*E. coli* (*n* = 11)	0	1 (9.1)	2 (18.2)	1 (9.1)	3 (27.3)	1 (9.1)	1 (9.1)	0	2 (18.2)	8 (72.7)
*K. pneumonia* (*n* = 9)	2 (22.2)	2 (22.2)	0	0	0	1 (11.1)	2 (22.2)	1 (11.1)	1 (11.1)	5 (55.6)
*K. oxytoca* (*n* = 5)	0	0	0	0	0	3 (60.0)	1 (20.0)	1 (20.0)	0	5 (100)
*C. jillenii* (*n* = 2)	0	0	0	0	0	0	1 (50.0)	1 (50.0)	0	2 (100)
*P. valgaris* (*n* = 1)	0	0	0	1 (100)	0	0	0	0	0	1 (100)
*P. stuartii* (*n* = 1)	0	0	1 (100)	0	0	0	0	0	0	0
*M. morgarin* (*n* = 1)	0	0	0	1 (100)	0	0	0	0	0	1 (100)
Total n (%)	2 (3.6)	3 (5.4)	5 (8.9)	5 (8.9)	5 (8.9)	12 (21.4)	13 (23.2)	8 (14.3)	3 (5.4)	46 (82.14)

Key: R0: sensitive for all classes of antibiotics; R1: resistance for one class of antibiotics; R2: resistance for two classes of antibiotics; R3: resistance for three classes of antibiotics; R5: resistance for five classes; R6: resistance for six classes of antibiotics: R7: resistance for seven classes of antibiotics: R8: resistance for eight classes of antibiotics

Abbreviations; CoNS: coagulase negative staphylococci, MDR: multiple drug resistance

### Prevalence and distribution of bacterial among cancer patients

In this study, bacterial cultures were positive in 56 (21.1%) patients (95% CI = 16.2–25.9) with 10 type of bacterial isolates identified. Susceptibility was higher among female participants, 30 (11.3%) compared to male, 26 (9.8%). The prevalence was higher, 18 (6.8%) among age range between 36 and 45 years and among rural residents, 33 (12.4%). A higher prevalence, 30 (11.3%) was also observed in participants with a monthly income of 1501–3000 ETB. Inpatient cases were more likely to develop bacterial infections, 38 (14.3%), with breast cancer patients showing the highest rate, 12 (4.5%). The prevalence was higher among patients receiving chemotherapy 42 (15.8%) and those with stage IV cancer level, 27 (10.2%). Patients with progressive cancer disease were susceptible to bacterial infection, 44 (16.5%) (Table 6).

**Table 6:** Bivariate and multivariable logistic regression analysis of factors associated with cancer patients at HUCSH, 2024

Socio-demographic variables	Categories	Culture growth *n* (%)		COR (95% CI)	*P*-value	AOR (95% CI)	*P*-value
		Positive	Negative				
Sex	Male	26 (9.8)	95 (35.7)	1			
	Female	30 (11.3)	115 (43.2)	1.05 (0.581-1.895)	0.874	-	
Age	< 25	2 (0.8)	11(4.1)	1			
	25–35	9 (3.4)	34 (12.8)	0.69 (0.128-3.672)	0.661	-	
	36–45	18 (6.8)	48 (18.0)	0.49 (0.098-2.404)	0.376	-	
	46–60	19 (7.1)	77 (28.9)	0.74 (0.151-3.606)	0.706	-	
	> 60	8 (3.0)	40 (15.0)	1		-	
Residence	Urban	23 (8.6)	106 (39.8)	1		1	
	Rural	33 (12.4)	104 (39.1)	0.68 (0.376-1.242)	0.212	0.47 (0.237-0.918)	0.027*
Marital status	Single	3 (1.1)	22 (8.3)	0.92 (0.083-10.140)	0.943	-	
	Married	52 (19.5)	180 (67.7)	0.43 (0.053-3.539)	0.435	-	
	Divorced	1 (0.4)	8 (3.0)	1			
Education status	No formal	27 (10.2)	90 (33.8)	0.93 (0.314-2.727)	0.889	-	
	Elementary	10 (3.8)	39 (14.7)	1.08 (0.323-3.633)	0.897	-	
	High school	14 (5.3)	63 (23.7)	1.25 (0.397-3.938)	0.703	-	
	Diploma & above	5 (1.9)	18 (6.8)	1			
Occupation type	Government	5 (1.9)	19 (7.1)	1			
	Private	12 (4.5)	34 (12.8)	0.75 (0.228-2.438)	0.627	-	
	Farmer	14 (5.3)	50 (18.8)	0.94 (0.298-2.967)	0.916	-	
	House hold	20 (7.5)	72 (27.1)	0.95 (0.315-2.854)	0.923	-	
	No work	5 (1.9)	35 (13.2)	1.84 (0.473-7.174)	0.378	-	
Monthly income	> 4500	7 (2.6)	37 (13.9)	0.80 (0.232-2.767)	0.726	0.91 (0.250-3.329)	0.890
	< 1500	14 (5.3)	42 (15.8)	0.46 (0.149-1.391)	0.167	0.51 (0.159-1.642)	0.260
	1501–3000	30 (11.3)	98 (36.8)	0.50 (0.177 − 1.380)	0.179	0.46 (0.156-1.349)	0.157
	3001–4500	5 (1.9)	33 (12.4)	1		1	
Smoking	Yes	1(0.4)	9 (3.4)	1			
	No	55 (20.7)	201 (75.6)	0.41 (0.050-3.274)	0.397	-	
Family history of cancer	Yes	3 (1.1)	8 (3.0)	1			
	No	53 (19.9)	202 (75.9)	1.43 (0.366-5.574)	0.607	-	
History of hospitalization	Yes	11 (4.1)	28 (10.5)	1		1	
	No	45 (16.9)	182 (68.4)	1.59 (0.736-3.432)	0.239	1.91 (0.805-4.506)	0.142
Patient setting	Inpatient	38 (14.3)	123 (46.2)	0.67 (0.359 − 1.250)	0.208	0.49 (0.229 − 1.030)	0.060
	Outpatient	18(6.8)	87 (32.7)	1		1	
Type of cancer	Breast	12 (4.5)	61 (22.9)	1.27 (0.130-12.388)	0.837	-	
	Colon	1 (0.4)	17 (6.4)	4.25 (0.216-83.517)	0.341	-	
	Gall bladder	4 (1.5)	9 (3.4)	0.56 (0.047-6.769)	0.650	-	
	Cervical	4 (1.5)	19 (7.1)	1.19 (0.103-13.654)	0.890	-	
	Esophageal	7 (2.6)	19 (7.1)	0.68 (0.064-7.161)	0.747	-	
	Pancreatic	2 (0.8)	9 (3.4)	1.13 (0.078-16.307)	0.931	-	
	Gastric	9 (3.4)	24 (9.0)	0.67 (0.065-6.793)	0.732	-	
	Hematology	2 (0.8)	3 (1.1)	0.38 (0.022-6.348)	0.497	-	
	Lung	4 (1.5)	5 (1.9)	0.31 (0.024-4.024)	0.372	-	
	Others	10 (3.8)	40 (15.0)	1.00 (0.100-9.957)	1.000	-	
	Prostatic	1 (0.4)	4 (1.5)	1			
Stage of cancer	I	5 (1.9)	8 (3.0)	0.11 (0.011-1.158)	0.066	0.08 (0.007-0.884)	0.039*
	II	15 (5.6)	41 (15.4)	0.20 (0.024-1.616)	0.130	0.11 (0.011-1.011)	0.051
	III	8 (3.0)	47 (17.7)	0.420 (0.048-3.649)	0.431	0.27 (0.028-2.577)	0.255
	IV	27 (10.2)	100 (37.6)	0.27 (0.033-2.102)	0.209	0.20 (0.023-1.795)	0.152
	Unknown	1(0.4)	14 (5.3)	1		1	
Cancer treatment started	Yes	45 (16.9)	159 (58.9)	0.76 (0.367-1.583)	0.466	-	
	No	11 (4.1)	51 (19.2)	1			
Type of treatment	Surgery	3 (1.1)	24 (9.0)	1		1	
	Chemo	42 (15.8)	149 (56.0)	0.44 (0.127-1.545)	0.202	0.54 (0.134-2.151)	0.379
	Follow up	11 (4.1)	37 (13.9)	0.42 (0.106-1.665)	0.217	0.42 (0.091-1.922)	0.263
Duration of therapy	≥ 1 year	4 (1.5)	23 (8.6)	1			
	< 1 year	41 (15.4)	150 (56.4)	0.64 (0.208-1.943)	0.427	-	
	Follow up	11 (4.1)	37 (13.9)	0.59 (0.166-2.056)	0.403	-	
History of surgery	Yes	13 (4.9)	53 (19.9)	1			
	No	43 (16.2)	157 (59.0)	0.90 (0.447-1.793)	0.755	-	
History of catheterization	Yes	7 (2.6)	18 (6.8)	1			
	No	49 (18.4)	192 (72.2)	1.52 (0.603-3.853)	0.374	-	
Duration of catheter	Previous	2 (0.8)	9 (3.4)	1			
	Current	5 (1.9)	9 (3.4)	0.40 (0.061-2.627)	0.340	-	
	No catheter	49 (18.4)	191 (72.2)	0.87 (0.182 − 4.160)	0.862	-	
Febrile	Yes	4 (1.5)	15 (5.6)	1			
	No	52 (19.5)	195 (73.3)	1.00 (0.318-3.141)	0.1000	-	
Cancer progression	Yes	44 (16.5)	179 (67.3)	1.58 (0.749-3.312)	0.231	2.52 (1.011–6.298)	0.047*
	No	12 (4.5)	31 (11.7)	1		1	

Key; AOR: Adjusted odds ratio, COR: Crude odds ratio, CI: Confidence interval

### Factors associated with culture positivity rate

In the bivariate analysis, patient residence, monthly income, patient setting, previous hospitalization, stage of cancer, type of treatment, and cancer progression were found to be statistically significant at a p-value of < 0.25 and were therefore considered as potential candidates for multivariate analysis. After adjusting for confounding variables, patients residing in rural areas were 47% less likely to be culture-positive for bacterial infection compared to urban residents (AOR = 0.47; 95% CI = 0.237–0.918; *P* = 0.027). Patients with stage I cancer were 8.0% less likely to develop bacterial infection compared to those with an unknown stage (AOR = 0.08; 95%CI = 0.007–0.884, *P* = 0.039). In addition, patients with progressive cancer were 2.52 times more likely to develop bacterial infection compared to those without progression (AOR = 2.52; 95%CI = 1.011–6.298, *P*=0.047) (Table 6).

## Discussion

Infection is one of the leading causes of morbidity and mortality among cancer patients, primarily due to cancer-related immune impairment and the immunosuppressive effects of its treatment [39]. In this study, the overall prevalence of culture-positive bacterial infection among cancer patients was 21.1%, with 95% CI = 16.2–25.9. This finding was comparable to studies conducted in other regions of Ethiopia reporting, 19.4% in Gondar [11] and 20.6% in Addis Ababa [40], as well as study from abroad, 24% in Nepal [8]. However, the observed prevalence in our study was higher than that reported in Romania 14.9% [41], Korea, 15% [42] and Indian, 4.6% [43]. In contrast, the result was lower than findings from another study in Ethiopia, 27% in Gondar [24], and 31.6% in Addis Ababa [44]. Such variations in prevalence across studies may be attributed to differences in study population characteristics, geographical distribution, sample size, sampling techniques, culturing methods, laboratory procedures, and diagnostic approaches [24, 45]. Additionally, hospital-related factors such as infection control practices, antibiotic stewardship programs, and the prevalence of nosocomial bacteria may also contribute to these differences [46].

In the current study, out of 56 bacterial isolates, Gram-negative bacteria (GNB) accounted for 54.0%, which was higher than Gram-positive bacteria. This finding is consistent with a study conducted in Gondar, Ethiopia [11], where GNB comprised 58.1%. However, it is relatively lower than reports from India, 69.93% [43] and Nepal 69.9% [20]. In contrast, the prevalence was higher than that reported in Palestine, 39.7% [47]. The predominance of GNB infections among cancer patients may be explained by cancer-related immunosuppression and the translocation of gut flora due to mucositis. It is also associated with the frequent use of broad-spectrum antibiotics in cancer patients, which can suppress Gram-positive flora and promote GNB overgrowth [20, 43]. Furthermore, prolonged hospitalization and the use of invasive devices such as catheters increase the risk of nosocomial GNB infections [48]. In addition, the production of extended-spectrum β-lactamase (ESBL) and carbapenemase enzymes by Enterobacteriaceae enables them to hydrolyze broad-spectrum antibiotics, contributing to drug resistance and prolonged colonization in hosts [49–51].

Coagulase-negative staphylococci (CoNS) accounted for 30.1% and were the most frequent bacterial isolates in the study. The prevalence of CoNS in this study was higher than that reported in Gondar, Ethiopia, 26.2% [11], and in Addis Ababa, Ethiopia, 6.7% [40], and in India study, 3.1% [52]. In contrast, the result was lower than findings from Addis Ababa, Ethiopia, 39.5% [23] and Palestine, 56.1% [2] studies. The high prevalence of CoNS among cancer patients can be attributed to several factors. Cancer patients are particularly susceptible to CoNS, a commensal skin flora, due to chemotherapy-induced immunosuppression and the frequent use of implantable devices such as prosthetics and central venous catheters [53, 54]. CoNS is also a common nosocomial pathogen, often acquired from the hospital environment as a result of frequent visits and prolonged hospitalization [55, 56]. Moreover, its ability to produce biofilms enhances survival on medical devices, contributing to chronic infections and treatment failure among cancer patients [54].

*E. coli* was the most prevalent isolate among Gram-negative bacteria (GNB), accounting for 19.6%. This prevalence was higher than that reported in studies from India (11.1%) [52] and Bihar, India (14.7%) [43], but lower than findings from Gondar, Ethiopia (21.4%) [11], Addis Ababa, Ethiopia (42.6%) [40], Palestine (30.3%) [2], Egypt (22.2%) [57], and Cairo, Egypt (36%) [6].

Similarly, the prevalence of *S. aureus* (8.9%) was higher than studies conducted in Addis Ababa, Ethiopia (6.7%) [40], Palestine (7.0%) [2], India (7.8%) [52]. In contrast, it was lower than reports from Gondar, Ethiopia (28.6%) [11] and other studies in India (13.72%) [43].

In the current study, other bacterial species such as *K. pneumoniae* (16.1%), *K. oxytoca* (8.9%), *C. jillenii* (3.6%), *P. vulgaris* (1.8%), *P. stuartii* (1.8%), and *M. morganii* (1.8%) were also identified. Although some species were not fully characterized, comparable organisms have been proportionally reported in other studies: Cairo, Egypt (*K. pneumoniae*, 30.85%) [6], Addis Ababa, Ethiopia (*K. pneumoniae*, 10.7% %) [23], Egypt (*M. morganii*, 3.8%, *P. stuartii*, 1.6%, Citrobacter spp., 3.5%) [57], Addis Ababa, Ethiopia (Klebsiella spp. 10.7%, Proteus spp. 9.3%, Citrobacter spp. 4%) [40], India (Klebsiella spp. 18.3%) [43], and Nepal (Klebsiella spp. 10%, Citrobacter spp. 8.0%, Proteus spp. 1.0%) [20]. *K. oxytoca* was most frequently reported in studies from Egypt (1.9%) [57], Bihar, India (10.7%) [52], and Jimma, Ethiopia (11.5%) [22].

Variations in the distribution of bacterial isolates across studies may be explained by differences in study populations, participants’ health conditions and lifestyles, geographical settings, socio-economic factors, seasonal variations, specimen types, and sample sizes [21, 58]. Additionally, differences in antibiotic use, infection control practices, and the implementation of antibiotic stewardship programs contribute to the observed disparities in pathogen distribution across countries [59].

Regarding antimicrobial sensitivity in this study, CoNS, the dominant organism, showed 88.9% resistance to nitrofurantoin, 72.2% to trimethoprim/sulfamethoxazole, and 38.9% to clindamycin. These rates were higher than those reported in Gondar, Ethiopia, where CoNS were fully sensitive to nitrofurantoin and showed 45.5% resistance to trimethoprim/sulfamethoxazole [11].

*E. coli* demonstrated marked resistance to ampicillin (90.0%) and tetracycline (81.8%), exceeding rates observed in Gondar, Ethiopia (ampicillin 77.8%) 11), Egypt (tetracycline 84.6%) [57], India (ampicillin 80.5%) [2], and Palestine (ampicillin 90.5%) 47). Conversely, *E. coli* resistance to meropenem (9.1%) and to gentamicin, chloramphenicol, and amikacin (18.2%) was relatively low. In Gondar, Ethiopia [11], *E. coli* resistance to amikacin was 11.8%, lower than our finding, while gentamicin resistance was 22.2%, higher than ours. Compared with India and Palestine, our results were relatively high, as *E. coli* in those studies showed no resistance to meropenem or amikacin [2, 47]. However, the Egyptian study reported 50.5% resistance to meropenem, exceeding our result [57].

*S. aureus* also showed 100% resistance to Trimethoprim/sulfamethoxazole, tetracycline, penicillin and cefoxitin, and 100% sensitivity to erythromycin. These results were higher than those reported in Palestine [47], where no resistance to trimethoprim/sulfamethoxazole or tetracycline was observed, and 40% resistance to erythromycin was documented. In Gondar, Ethiopia [11], resistance rates were 41.7% for trimethoprim/sulfamethoxazole, 60% for tetracycline, and 58.3% for penicillin, while in India tetracycline resistance was 50.0% [2].

Moreover, 46 (82.1%) of bacterial isolates in this study were MDR. This result was relatively higher than previous reports from Ethiopia, Addis Ababa (33.3%) [10] and Gondar (46.5%) [11], as well as from other countries such as Argentina (42.3%) [60], the United States (48%) [61], and the ALARICO report, which reported 66.9% of cancer patients with bloodstream infections having MDR Gram-negative bacteria [17]. However, our result is closest to the report from India (74.1%) [62], and lower than a previous Ethiopian study in Jimma, which detected 88.3% MDR GNB [22].

The observed variation in antibiotic resistance and the high phenotypic MDR among isolates could primarily be attributed to prolonged cancer treatment with chemotherapy, the use of prophylactic drugs, and the empirical administration of antibiotics in cancer patients. Drug resistance and reduced drug efficacy in this population are also linked to specific conditions such as hypoxia and autophagy within cancer cells [63]. Moreover, the emergence of MDR may result from extended-spectrum β-lactamase and carbapenemase production by Enterobacteriaceae, which harbor resistance genes and modify antimicrobial targets [49, 50]. In addition, many cancer patients are admitted to healthcare facilities, where they are exposed to and acquire drug-resistant pathogens [14].

The majority of our samples were urine specimens from UTI cases, in which gut-associated Enterobacteriaceae are the predominant pathogens. These organisms may have adapted to prophylactic drug exposure [64], potentially contributing to the higher MDR rates observed compared with other studies. Non-compliance with prescribed regimens and excessive drug consumption in the study area may also play a role [65, 66]. Finally, differences in sample type, laboratory methods for bacterial isolation, identification, and susceptibility testing could further explain the discrepancies observed across studies.

In the multivariate analysis of associated factors, being a rural resident (AOR = 0.47; 95% CI = 0.237–0.918; *P* = 0.027) and stage I cancer (AOR = 0.08; 95%CI = 0.007–0.884; *P* = 0.039) were associated with a 47% and 8.0% lower likelihood, respectively, of developing bacterial infection compared with urban residents and patients with unknown cancer stage. In contrast, patients with progressing cancer had a 2.52 fold increased risk of bacterial infection compared with those without disease progression (AOR = 2.52; 95%CI = 1.011–6.298, *P*=0.047). The adjusted odds ratios for rural residence should be interpreted relative to urban populations, highlighting disparities in infection risk between these settings. Similarly, odds ratios for each cancer stage are interpreted relative to patients with unknown stage, which frames the findings in the context of incomplete staging data. While this choice preserves sample size, it may limit direct clinical comparability, and we acknowledge this as a methodological consideration. Reports from Palestine showed that bacterial infections were strongly associated with hospital-acquired infections [59].

Urban populations are generally more prone to bacterial infections than rural populations due to factors such as higher population density, increased mobility, and greater interpersonal contact. Access to healthcare facilities, while beneficial, also increases exposure to healthcare-associated pathogens, and environmental conditions such as poor sanitation further facilitate disease transmission [67, 68]. Patients with stage I cancer are less likely to develop infections because their disease is typically localized, non-metastatic, and less likely to compromise immune function [69]. Moreover, most stage I patients are not yet exposed to intensive immunosuppressive treatments such as chemotherapy and radiation [70]. By contrast, patients with progressive cancer are more susceptible to infection due to treatment-related immunosuppression from radiation and cytotoxic drugs, which can result in neutropenia, mucositis, and skin toxicity [71], as well as tumor-related and iatrogenic immunosuppression [72].

### Limitation of study

The limitation of this study is the absence of molecular or genotypic characterization of antimicrobial resistance, including confirmation of multidrug resistance. This was primarily due to financial constraints and limited laboratory capacity. As a result, resistance patterns were assessed using phenotypic methods alone. While these methods provide valuable insights, the lack of molecular confirmation may restrict the depth of interpretation and limit the generalizability of the findings to broader contexts.

## Conclusion

In the current study, the prevalence of bacterial infection among cancer patients was high (21.1%). CoNS and *E. coli* were the predominant causative agents. A high level of resistance to commonly used antibiotics including nitrofurantoin, ampicillin, penicillin, and tetracycline was observed, and a notable prevalence of MDR isolates was also detected. However, meropenem, amikacin, and chloramphenicol were the most effective antibiotics against Gram-negative bacterial infections, while clindamycin and erythromycin were more effective against Gram-positive bacterial infections among cancer patients. Rural residence, stage I cancer, and progressive cancer showed significant associations with bacterial infections in cancer patients. Therefore, treating bacterial infections in cancer patients should rely on antimicrobial susceptibility testing. Further research on antimicrobial resistance, together with strong antibiotic stewardship and infection control measures, is required. Additionally, identifying risk factors and monitoring resistance trends are critical for managing infections and mitigating drug resistance among cancer patients, particularly at the study site.

## Supplementary Information

Below is the link to the electronic supplementary material.

Supplementary Material 1

## Data Availability

The datasets used and/or analyzed during the current study are available from the corresponding author at reasonable request.

## Abbreviations

HUCSH: Hawassa University Comprehensive Specialized Hospital
IRB: Institutional Review Board
MDR: Multidrug resistance
