# Supplementary material for: Bacterial profile, antimicrobial resistance patterns and associated factors among cancer patients at Hawassa University Comprehensive Specialized Hospital, Sidama region, Southern Ethiopia
Source: BMC Infect Dis. 2026 Apr 10;26:1000. doi: 10.1186/s12879-026-13061-8 (PMC13196089; doi:10.1186/s12879-026-13061-8)
Supplement: Supplementary file 1 — Supplementary Material 1 [file 12879_2026_13061_MOESM1_ESM.docx]

**Hawassa University Comprehensive Specialized Hospital, Cancer Center**

**Consent form before conducting an interview**

**Questionnaire**: To determine the bacterial profile, antimicrobial resistance patterns, and factors associated among cancer patients at Hawassa University Comprehensive Specialized Hospital, Sidama region, Southern Ethiopia.

Hello!! Good morning/good afternoon? I am __________________. I am a data collector for the research to be conducted by Miss Alemitu Beyene (PI) the instructor and researcher of Clinical Microbiologist at Hawassa University, College of Medicine and Health Science. She is conducting study on the Bacterial Profile, Antimicrobial Resistance Patterns and Factors Associated of Cancer Patients, at this clinic from January 1 to June 30, 2024. The objective of this interviewing is to collect socio-demographic and clinical data from cancer patients to determine the bacterial profile, antibiotics susceptibility patterns and associated factors of bacterial infection suspected cancer patients at Hawassa University Comprehensive Specialized Hospital. As a chance, you are selected to be part of the study by a chance. It will take about 15-20 minutes to interview questionnaire. If you are willing to participate in this study, you are kindly requested to give us the correct information about yourself. The required socio-demographic and clinical data, a blood or urine, sputum or swab samples will be collected by trained data collector’s nurses and physician. I would like to assure you; the information that you provide me is completely confidential and will be used only for the research purpose. You will not get or provide any incentive or payments that you are participating in the study rather it is full of voluntary and you can withdraw in the begging or middle of the interview if you not satisfy it. Considering the information, you get from the general information above, your role in the success of the research is important and I appreciate your contribution to the research.

Do you have any other questions? Would you agree and willing to participate in this study please? **1. Yes 2. No**

I understood the advantage of the research and the roles I will have in the research. I have agreed to participate in the research.

Signature (participant)­­­­­­­­­­­­­__________________ Date_____/_____/_________

Signature (data collector) ________________Date_____/_____/_________

Thank you for participation, if you want to know more information, you can contact the investigator of the research by the following address below.

**Miss Alemitu Beyene**

Hawassa University College of Medicine and Health Sciences

Cell phone: +251910187049, [***E-mail-alemitubeyene5@gmial.com***](mailto:E-mail-alemitubeyene5@gmial.com)

**For exclusion of participants**

[If he/she say ‘’yes’’ point listed below, he/she will be excluded from the study]

1. Patients who were on antibiotic treatment in the last 14 days

**Patient ID. ____________________________________ Date _____/_____/_________**

| **No** | **Inquiries** | **Response**  **(circle clients response)** |
| --- | --- | --- |
|  | Sex | - 1. Male   2. Female |
|  | How old are you? | _______years |
|  | Do you tell me your origin of residence? | 1. Rural 2. Urban |
|  | Do you tell me your marital status? | - 1. Single   2. Married   3. Divorced   4. Widower or Widower |
|  | Do you tell me your educational level? | - 1. No formal education   2. Elementary   3. High school (9-12)   4. Diploma & above |
|  | Do you tell me your occupation type? | 1. Government employee 2. Private employee 3. Farmer 4. House hold 5. Student 6. No work |
|  | Do you tell me your monthly income average? | _________birr (ETB) |
|  | Do you smoking cigarette? | - 1. Yes   2. No |
|  | **Clinical variables** |  |
|  | Is there cancer history in your family? | - 1. Yes   2. No |
|  | Have you history of previous hospitalization? | - 1. Yes   2. No |
|  | Patient setting | - 1. Inpatient   2. Outpatient |
|  | Type cancer | 1. Breast 2. Colon 3. Gall bladder 4. Cervical 5. Esophageal 6. Pancreatic 7. Gastric 8. Hematology 9. Lung 10. Prostatic 11. Others ________ |
|  | Stage of cancer | - 1. I   2. II   3. III   4. IV   5. Unknown |
|  | Have you started cancer treatment? | - 1. Yes   2. No |
|  | What types of cancer therapy did you started? | 1. Surgery 2. Chemotherapy 3. Follow up 4. Radiation 5. Immunotherapy |
|  | For how a much time you stay on this cancer therapy? | 1. ≥ 1 year 2. < 1 year 3. Follow up |
|  | Do you have any surgical incision history? | - 1. Yes   2. No |
|  | Do you have the history of catheterization? | - 1. Yes   2. No |
|  | When do you use the catheter? | - 1. Previous   2. Current |
|  | Febrile | - 1. Yes   2. No |
|  | Progression of cancer | - 1. Yes   2. No |
